# Zinc Inhibits HIF-Prolyl Hydroxylase Inhibitor-Aggravated VSMC Calcification Induced by High Phosphate

**DOI:** 10.3389/fphys.2019.01584

**Published:** 2020-01-15

**Authors:** Annamária Nagy, Dávid Pethő, Tamás Gáll, Erzsébet Zavaczki, Mónika Nyitrai, József Posta, Abolfazl Zarjou, Anupam Agarwal, György Balla, József Balla

**Affiliations:** ^1^Department of Internal Medicine, Faculty of Medicine, University of Debrecen, Debrecen, Hungary; ^2^Kálmán Laki Doctoral School, University of Debrecen, Debrecen, Hungary; ^3^HAS-UD Vascular Biology and Myocardial Pathophysiology Research Group, Hungarian Academy of Sciences, Debrecen, Hungary; ^4^Department of Pediatrics, Faculty of Medicine, University of Debrecen, Debrecen, Hungary; ^5^Department of Inorganic and Analytical Chemistry, UD Faculty of Science and Technology, University of Debrecen, Debrecen, Hungary; ^6^Nephrology Research and Training Center, School of Medicine, University of Alabama at Birmingham, Birmingham, AL, United States

**Keywords:** VSMC, vascular calcification, phosphate, Zn supplementation, Pi, prolyl hydroxylase inhibitor

## Abstract

Vascular calcification is a life-threatening clinical condition in chronic kidney disease (CKD) and is associated with reduced zinc serum levels. Anemia is another frequent complication of CKD. Hypoxia-inducible factor (HIF) stabilizers, also known as HIF prolyl hydroxylase inhibitors (PHI), are promising candidates to treat CKD-associated anemia by increasing erythropoietin synthesis. Recent evidence suggests that HIFs play a pivotal role in vascular calcification. Our study explored feasible impacts of HIF PHI on phosphate (Pi)-induced calcification of vascular smooth muscle cells (VSMCs) and tested whether zinc might inhibit this mineralization process. Treatment of VSMCs with PHI aggravated Pi-induced calcium deposition and Pi uptake. PHI promoted Pi-induced loss of smooth muscle cell markers (ACTA-2, MYH11, SM22α) and enhanced osteochondrogenic gene expression (Msx-2, BMP-2, Sp7) triggering osteochondrogenic phenotypic switch of VSMCs. These effects of PHI paralleled with increased pyruvate dehydrogenase kinase 4 (PDK4) expression, decreased Runx2 Ser451 phosphorylation, and reduced cell viability. Zinc inhibited Pi-induced mineralization of VSMCs in a dose-dependent manner and also attenuated the pro-calcification effect of PHI in Pi-induced mineralization. Zinc inhibited osteochondrogenic phenotypic switch of VSMCs reflected by lowering Pi uptake, decreasing the expressions of Msx-2, BMP-2, and Sp7 as well as the loss of smooth muscle cell-specific markers. Zinc preserved phosphorylation state of Runx2 Ser451, decreased PDK4 level, and restored cell viability. PHI alone reduced the expression of smooth muscle markers without inducing mineralization, which was also inhibited by zinc. In addition, we observed a significantly lower serum zinc level in CKD as well as in patients undergoing carotid endarterectomy compared to healthy individuals. Conclusion - PHI promoted the loss of smooth muscle markers and augmented Pi-induced osteochondrogenic phenotypic switch leading to VSMCs calcification. This mineralization process was attenuated by zinc. Enhanced vascular calcification is a potential risk factor during PHI therapy in CKD which necessitates the strict follow up of vascular calcification and zinc supplementation.

## Introduction

Anemia is a clinical hallmark of chronic kidney disease (CKD) representing a worldwide burden on public health (Levey et al., 2007; Webster et al., 2017) by reducing the quality of life and increasing cardiovascular disease and mortality (KDOQI and National Kidney Foundation, 2006). One of the main characteristics of systemic vascular pathology in chronic renal failure is the accelerated vessel wall calcification (Palit and Kendrick, 2014). There is a continuous and innovative evolution in the treatment of CKD-associated anemia, but preventive and therapeutic interventions against vascular calcification are still limited. A promising approach to correct CKD-associated anemia is the pharmacologic inhibition of hypoxia-inducible factor (HIF) prolyl-4-hydroxylase domain (PHD) proteins. HIFs regulate multiple genes, including erythropoietin (EPO) and EPO receptor, and proteins promoting iron absorption, iron transport (transferrin), and heme synthesis (Peyssonnaux et al., 2007; Kaelin and Ratcliffe, 2008). Hydroxylation of HIFs is performed by three PHD proteins PHD1, PHD2, and PHD3 (Schofield and Ratcliffe, 2004). Under normoxia, PHD proteins hydroxylate HIFs facilitating their polyubiquitination and proteasomal degradation, thereby inhibiting the activation of HIF-responsive genes (Fong and Takeda, 2008). Thus, pharmacological inactivation of PHD enzymes stimulates endogenous EPO production both in animals and humans (Hsieh et al., 2007; Bernhardt et al., 2010). FG4592 is one of the orally bioavailable HIF PHD inhibitors (PHI) promoting erythropoiesis through an HIF-dependent manner. Clinical trials have demonstrated that PHI FG4592 effectively corrects anemia in CKD patients (Besarab et al., 2015, 2016; Provenzano et al., 2016; Chen et al., 2017), therefore, together with other HIF PHIs, has gained attention as a promising alternative for erythropoiesis-stimulating agents (Gupta and Wish, 2017).

Chronic kidney disease and end-stage kidney disease are associated with increased cardiovascular mortality and morbidity (Sigrist et al., 2007; Collins et al., 2015). In CKD, the prevalence of vascular calcification is high and appears at a younger age compared to the healthy population (Goodman et al., 2000). Hyperphosphatemia is closely associated with advanced vascular calcification (Shigematsu et al., 2003; Adeney et al., 2009) and increases mortality in CKD (Kestenbaum et al., 2005). Clinical drugs against vessel wall calcification are still not available, but research effort is significant in exploring this field. It is documented that plasma zinc levels are low in dialysis patients and zinc supplementation has been shown to attenuate high phosphate induced calcification and osteoblastic transformation of VSMC (Shin and Kwun, 2014; Voelkl et al., 2018).

Recent evidence suggests that HIFs play a pivotal role in vascular calcification (Mokas et al., 2016; Zhu et al., 2018). It is uncovered whether HIF stabilizer PHIs have any effect on the mineralization of VSMC. PHI FG4592, one of the promising PHIs, prompted us to explore the influence of PHI FG4592 on vessel calcification using an *in vitro* approach. Since the level of plasma zinc is low in CKD, we sought to investigate the relationship between PHIs and zinc in vascular calcification. In this study, we provide evidence that PHI FG4592 facilitates phosphate induced calcification in VSMC, which is attenuated by zinc supplementations.

## Materials and Methods

### Reagents

All reagents were purchased from Sigma-Aldrich (St. Louis, MO, United States) unless otherwise stated.

### Cell Culture

Human aortic smooth muscle cells (VSMCs) were purchased from Cell Applications (San Diego, CA, United States, Cat.: 354-05a) and Lonza (Allendale, NJ, United States Cat.: CC-2571). Zinc dose experiments were performed on cells purchased from Cell Applications, while all the other experiments were performed on VSMCs purchased from Lonza. Cells were grown in Dulbecco’s Modified Eagle Medium (DMEM) containing 1000 mg/L glucose supplemented with 10% fetal bovine serum (Life Technologies, Vienna, Austria), 100 U/ml penicillin, 100 μg/ml streptomycin, and neomycin referring to as growth medium (GM). Cells were grown to confluence and used from passages 5 to 7. Media were changed every 2 days. To induce phosphate mediated calcification, GM was supplemented with inorganic phosphate (3 mmol/L referring to as calcification medium) in a form of Na_2_HPO_4_/NaH_2_PO_4_ (pH 7.4) in the presence or absence of zinc (15–30 μmol/L of ZnCl_2_ × 6H_2_O) for 10 days. To investigate the effect of PHI FG4592 on VSMC calcification, cells were treated with FG4592 (Selleckchem, Houston, United States) in various concentrations (5 and 20 μmol/L) in calcification medium in the presence or absence of zinc (30 μmol/L) for 3, 6 or 10 days.

### Human Samples

Healthy volunteers were recruited without any known diseases including hypertension, dyslipidemia, liver and kidney malfunctions (*n* = 18, mean age 46 years, F/M 7/11). Stage 5 CKD patients subjected to hemodialysis were selected (*n* = 28, mean age 55 years, F/M 15/13) from the dialysis unit of the Clinical Centre, Department of Internal Medicine, University of Debrecen, Debrecen, Hungary. Vascular disease group was selected from patients undergoing carotid endarterectomy (*n* = 15, mean age 72 years, F/M 6/9). Volunteers did not take any zinc supplementation. Blood was drawn in Vacutainers (BD, Franklin Lakes, NJ, United States) using citrate immediately before surgery or the hemodialysis session from CKD patients by venipuncture. Participants gave their informed consent to the study, which was approved by the Regional and Institutional Ethics Committee of the University of Debrecen, Medical and Health Science Center. All subjects gave written informed consent in accordance with the Declaration of Helsinki.

### Measurement of Plasma Zinc Concentration With Inductively Coupled Plasma-Atomic Emission Spectrometry (ICP-AES)

A five milliliters serum samples were digested two times with 10 – 10 ml Carlo Elba nitric acid 65% (m/m) on water-bath at atmospheric conditions. The digestion was finished with 2 – 2 ml analytical grade hydrogen peroxide 30% (m/m) (Reanal, Hungary). The digested samples were filled to 10 ml with deionized water. At the same time with these sample digestion, we prepared blank solutions from nitric acid and hydrogen peroxide, too. Flame atomic absorption spectrometric (FAAS) analysis was carried out using the following settings. Hollow cathode lamp: Zn Cathodeon lamp, Lamp current: 10 mA, Wavelength: 213.9 nm, Slot burner: 10 cm, Acetylene: 1 L/min, Air: 5 L/min (3 bar), Observation height: 10 mm. The zinc concentrations are the average of three parallel measurements.

### Alizarin Red Staining

Cells were washed with phosphate-buffered saline (PBS) pH 7.4 without Ca^2+^ and Mg^2+^ and fixed with 4% paraformaldehyde for 10 min at room temperature. Then, cells were rinsed with PBS without Ca^2+^ and Mg^2+^ and stained with 2% Alizarin Red S for 10 min. Excessive dye was removed by several washes in deionized water. Extracellular calcium deposition was stained in red color.

### Quantification of Calcium Deposition

Cells grown on 24-well plates were washed twice with PBS pH 7.4 without Ca^2+^ and Mg^2+^ and decalcified with 0.6 mol/L HCl for 30 min at 37°C. After decalcification, cells were solubilized with a solution of NaOH 0.1 mol/L and SDS 0.1%, and the protein content of samples was measured with bicinchoninic acid (BCA) protein assay kit (Pierce, Rockford, IL, United States). The calcium content of the cells was normalized to protein content and expressed as μg/mg protein. The calcium content of the supernatants was determined by the QuantiChrom Calcium Assay Kit (Gentaur, Brussels, Belgium).

### Measurement of Intracellular Inorganic Phosphate

Intracellular inorganic phosphate concentration was determined by colorimetric analysis using QuantiChrom^TM^ Phosphate Assay Kit (Gentaur). Cells were washed twice with PBS pH 7.4 without Ca^2+^ and Mg^2+^ and then lysed with solubilization buffer (1% Triton-X 100, 0.5% Igepal CA-630, 10% protease inhibitor. 150 mmol/L NaCl, 5 mmol/L EDTA, 10 mmol/L Tris). Intracellular phosphate concentration of the cells was normalized to protein content expressed as μmol/mg protein.

### RNA Isolation and Quantitative Reverse Transcription – Polymerase Chain Reaction

Vascular smooth muscle cells were grown on 24-well plates and total RNA was isolated using TRIzol (Invitrogen, Carlsbad, CA, United States). The cDNA was synthesized using a High-Capacity cDNA Reverse Transcription Kit (Applied Biosystems, Foster City, CA, United States) for RT-PCR. Real-time polymerase chain reactions were performed using fluorescent TaqMan probes. TaqMan gene expression assays for BMP-2 (Hs00154192_m1), Msx-2 (Hs00741177_m1), PDK-4 (Hs01037712_m1), TAGLN/SM22α (Hs0103877_g1), smooth muscle α-actin/ACTA2 (Hs00426895_g1, MYH11 (Hs00975796_m1), Sp7 (Hs01866874_s1), and RNA45S5 (Hs05627131_gH) were purchased from Thermo Scientific, United States. Gene expressions were normalized to RNA45S5 (Hs05627131_gH). Polymerase chain reactions were carried out using the iCycler iQ Real-Time PCR system (Bio-Rad, Hercules, CA, United States) and the results represent the relative mRNA expression of target genes normalized RNA45S5 mRNA levels.

### Preparation of Whole-Cell Lysates and Nuclear Fractions

To prepare whole cell lysates, VSMCs were washed with PBS pH 7.4 without Ca^2+^ and Mg^2+^ then lysed using RIPA buffer (50 mmol/L Tris [pH 7.5], 150 mmol/L NaCl, 1% Igepal CA-630, 1% sodium deoxycholate, 0,1% SDS) containing 10% Complete Mini Protease Inhibitor Cocktail and 20% PhosSTOP phosphatase inhibitor cocktail, and incubated for 20 min on ice. Lysates were centrifuged at 14,000 × *g*, 4°C for 20 min. Supernatants were used as whole-cell extracts.

For nuclear fraction separation, cells were washed with PBS pH 7.4 without Ca^2+^ and Mg^2+^ then scraped with ice-cold hypotonic buffer (20 mmol/L HEPES, 250 mmol/L Sucrose, 10 mmol/L KCl, 2 mmol/L MgCl_2_) containing 10% Complete Mini Protease Inhibitor Cocktail and 20% PhosSTOP phosphatase inhibitor cocktail on ice. Using 1 ml syringe, the cell suspension was passed through a 28G needle 30 times then swelled on ice for 20 min. Lysates were centrifuged at 15,000 × g, 4°C for 15 min. Supernatants were discarded and this procedure was repeated 4 times. Finally, the nuclear pellets were dissolved in RIPA buffer supplemented with 10% Complete Mini Protease Inhibitor Cocktail and 20% PhosSTOP phosphatase inhibitor cocktail and centrifuged at 16,000 × *g*, 4°C for 15 min. Supernatants were used as nuclear fractions. Protein content was determined using the bicinchoninic acid assay (Pierce BCA Protein Assay Kit, Thermo Fisher Scientific, Waltham, MA, United States).

### Immunoblotting

Cell extracts (25–30 μg protein) were electrophoresed on 10 or 12% Tris–glycine SDS-gels and proteins were transferred to 0.22 μm PVDF membrane (Advansta Inc., Menlo Park, CA, United States). Membranes were blocked with 5% w/v milk for 60 min at room temperature. After blocking, membranes were incubated with Runx-2 antibody (Cell Signaling Technology, Danvers, MA, United States, Cat.: 12556) at 1:1000 dilution, Runx-2 (Ser 451) antibody (Bioss Antibodies Inc., Woburn, MA, United States, Cat.: bs-5685R) at 1:500 dilution, metallothionein (Abcam, Cambridge, United Kingdom; Cat.: # ab12228) 1:1000, and PDK-4 (Abcam, Cat.: ab88063) at 1:1000 dilution. Antigen-antibody complexes were visualized with the horseradish peroxidase chemiluminescence system (Advansta, Menlo Park, CA, United States). Protein bands were normalized to Hsp90 (Cell Signaling; Cat.: 4874S and Lamin B1 (Proteintech, Manchester, United Kingdom, Cat: 66095-I-Ig).

### MTT Assay

Cell viability was determined by the MTT assay. Briefly, cells were cultured and treated in 96-well plates for the indicated time. Then cells were washed with PBS, and 100 μl of 3-[4,5-dimethylthiazol-2-yl]-2,5-diphenyl-tetrazolium bromide (0.5 mg/ml) solution in HBSS was added. After a 90-minute incubation, the MTT solution was removed, formazan crystals were dissolved in 100 μL of DMSO and optical density was measured at 570 nm.

### MT1a and MT2a siRNA Transfection

Small interfering RNA specific to MT1a (Cat.: 4392420; ID: s194620) and MT2a (Cat.: 4392420; ID: s194629) and negative control siRNA (Cat.: 4390843) were obtained from Ambion (Thermo Scientific, Waltham, MA, United States). Transfection of siRNA into VSMCs was performed using Oligofectamine reagent (Thermo Scientific, Waltham, MA, United States) according to the manufacturer’s guide. Briefly, the cells were plated on 24-well plates to form 60–70% confluent monolayers. MT1a and MT2a siRNA were applied at 20 pmol/L concentration and transfection reagent complex were added to the cells for 5 h in OPTI-MEM Reduced Serum Medium then replaced to the growth medium for 16 h then to calcification or normal growth medium until the next transfection procedure. This transfection protocol was repeated every 3 days. Gene expression of MTs was analyzed by q-RT-PCR and immunoblot after 10 days.

### Statistical Analysis

Data are shown as mean ± SEM. Statistical analysis was performed by one-way ANOVA test followed by Bonferroni correction. A value of *p* < 0.05 was considered significant.

## Results

### Zinc Inhibits Phosphate-Induced Mineralization of VSMCs in a Dose-Dependent Manner

**FIGURE 1:**
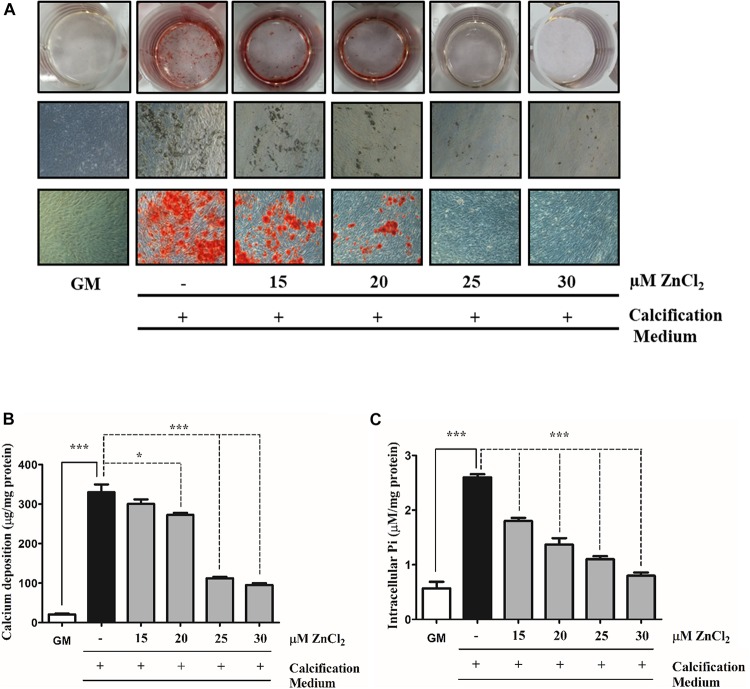
Zinc inhibits phosphate induced mineralization of VSMCs in a dose-dependent manner. VSMCs were cultured in calcification medium containing 3 mmol/L phosphate (Pi) in the presence (15–30 μmol/L) or absence of zinc for 10 days **(A–C)**. **(A)** Calcium deposition as a marker of ECM mineralization was visualized by Alizarin Red staining. Representative wells and images of stained plates from three independent experiments are shown. Statistical analysis was performed by one-way ANOVA test followed by Bonferroni correction. A value of *p* < 0.05 was considered significant. ^∗^*p* < 0.05, ^∗∗^*p* < 0.01, ^∗∗∗^*p* < 0.001. **(B)** The calcium content of solubilized ECM is presented. Data are expressed as mean ± SEM of three independent experiments. Statistical analysis was performed by one-way ANOVA test followed by Bonferroni correction. A value of *p* < 0.05 was considered significant. ^∗^*p* < 0.05, ^∗∗^*p* < 0.01, ^∗∗∗^*p* < 0.001. **(C)** Intracellular Pi levels were determined from the cell lysates and results are presented as mean ± SEM of three independent experiments. Statistical analysis was performed by one-way ANOVA test followed by Bonferroni correction. A value of *p* < 0.05 was considered significant. ^∗^*p* < 0.05, ^∗∗^*p* < 0.01, ^∗∗∗^*p* < 0.001. Experiments were performed on VSMCs purchased from Cell Applications.

**FIGURE 2:**
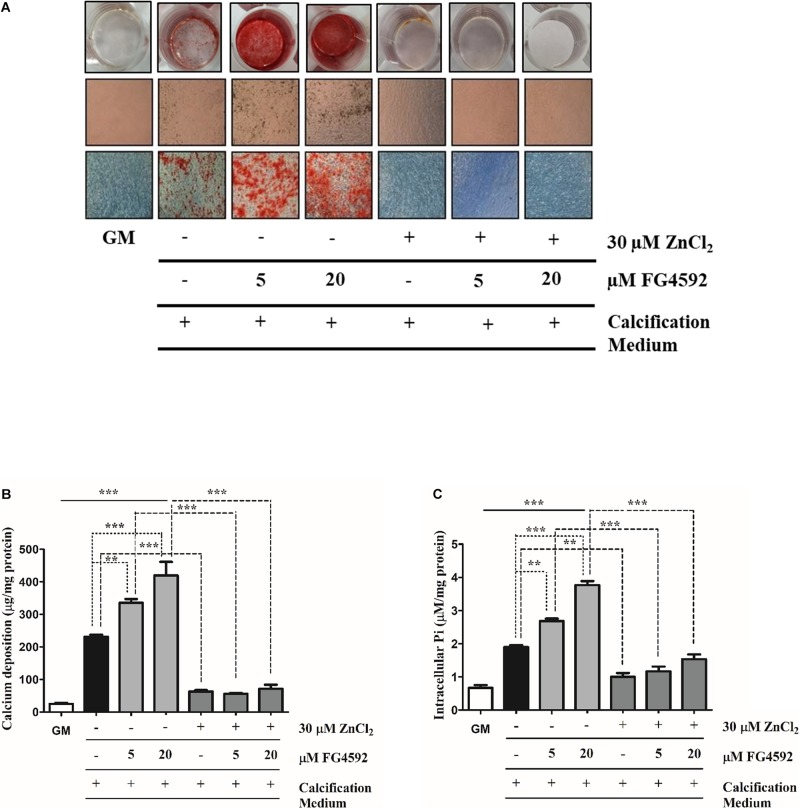
Prolyl hydroxylase inhibitor (PHI) FG4592 enhances Pi-induced mineral deposition in VSMCs that is inhibited by zinc. VSMCs cultured in calcification medium were treated with PHI FG4592 (5 and 20 μmol/L, respectively) in the presence (30 μmol/L) or absence of zinc for 10 days **(A–C)**. **(A)** Calcium deposition as a marker of ECM mineralization was visualized by Alizarin Red staining. Representative wells and images of stained plates from three independent experiments are shown. Statistical analysis was performed by one-way ANOVA test followed by Bonferroni correction. A value of *p* < 0.05 was considered significant. ^∗^*p* < 0.05, ^∗∗^*p* < 0.01, ^∗∗∗^*p* < 0.001. **(B)** The calcium content of solubilized ECM is presented. Data are expressed as mean ± SEM of three independent experiments. Statistical analysis was performed by one-way ANOVA test followed by Bonferroni correction. A value of *p* < 0.05 was considered significant. ^∗^*p* < 0.05, ^∗∗^*p* < 0.01, ^∗∗∗^*p* < 0.001. **(C)** Intracellular Pi levels were determined from the cell lysates and results are presented as mean ± SEM of three independent experiments. Statistical analysis was performed by one-way ANOVA test followed by Bonferroni correction. A value of *p* < 0.05 was considered significant. ^∗^*p* < 0.05, ^∗∗^*p* < 0.01, ^∗∗∗^*p* < 0.001.

Zinc has been shown to prevent phosphate (Pi)-induced osteogenic *trans*-differentiation of VSMCs (Shin and Kwun, 2014, 2016). To examine whether zinc inhibits Pi-provoked mineralization of VSMCs in our experimental conditions, we cultured human VSMCs in calcification medium containing 3 mmol/L inorganic Pi in the presence (15–30 μmol/L) or absence of zinc for 10 days (Figures 1A–C). Granular deposits were examined by Alizarin Red Staining which revealed that Pi significantly promoted mineral deposition compared to the untreated cells, while the exposure of VSMCs to zinc dose-dependently decreased Pi-induced mineralization (Figure 1A) and significantly decreased extracellular matrix (ECM) calcium content (Figure 1B). Active uptake of Pi into the cells *via* the type III sodium-dependent phosphate cotransporter, Pit-1, is a key factor of Pi-induced calcification of VSMCs (Li et al., 2006). Therefore, we measured Pi uptake by analyzing intracellular Pi levels in calcification medium in the presence or absence of zinc. Pi induced a robust increase in intracellular Pi compared to untreated cells, while at a concentration of 15 μmol/L, zinc significantly lowered Pi uptake (Figure 2C). Importantly, 30 μmol/L zinc decreased Pi content of VSMCs exposed to high Pi down to the level of cells maintained in growth medium (Figure 2C). Overall, these data demonstrate that zinc effectively inhibits Pi-induced mineral deposition and Pi uptake by human VSMCs in a dose-dependent manner.

### PHI Enhances Pi-Induced Mineral Deposition in VSMCs That Is Inhibited by Zinc

Hypoxia greatly enhances VSMCs calcification and osteochondrogenic *trans*-differentiation (Mokas et al., 2016) which raises the question whether stabilization of HIFs influences VSMCs calcification. VSMCs cultured in calcification medium were exposed to PHI FG4592 (5 and 20 μmol/L, respectively) in the presence (30 μmol/L) or absence of zinc for 10 days, and mineral deposition, the calcium content of ECM as well as Pi uptake by VSMCs were analyzed (Figures 2A–C). We showed that PHI markedly enhanced Pi-induced VSMC mineralization compared to Pi alone, while zinc (30 μmol/L) inhibited enhanced calcification by PHI (Figure 2A). To quantify the extent of matrix mineralization, the calcium content of the ECM was measured after 10 days. PHI increased the calcium content of the ECM in a dose-dependent manner compared to Pi alone, which was significantly attenuated when zinc (30 μmol/L) was present in the experimental medium (Figure 2B). Similar to ECM calcium content, PHI dose-dependently enhanced Pi uptake and intracellular Pi levels compared to high Pi alone that was markedly inhibited by zinc (Figure 2C). Overall, our data presented here show that chemical stabilization of HIF by PHI FG4592 strongly enhances Pi-induced mineralization and Pi uptake by VSMC, and these processes are significantly attenuated by zinc supplementation.

### PHI Decreases Smooth Muscle-Specific Marker Expression

**FIGURE 3:**
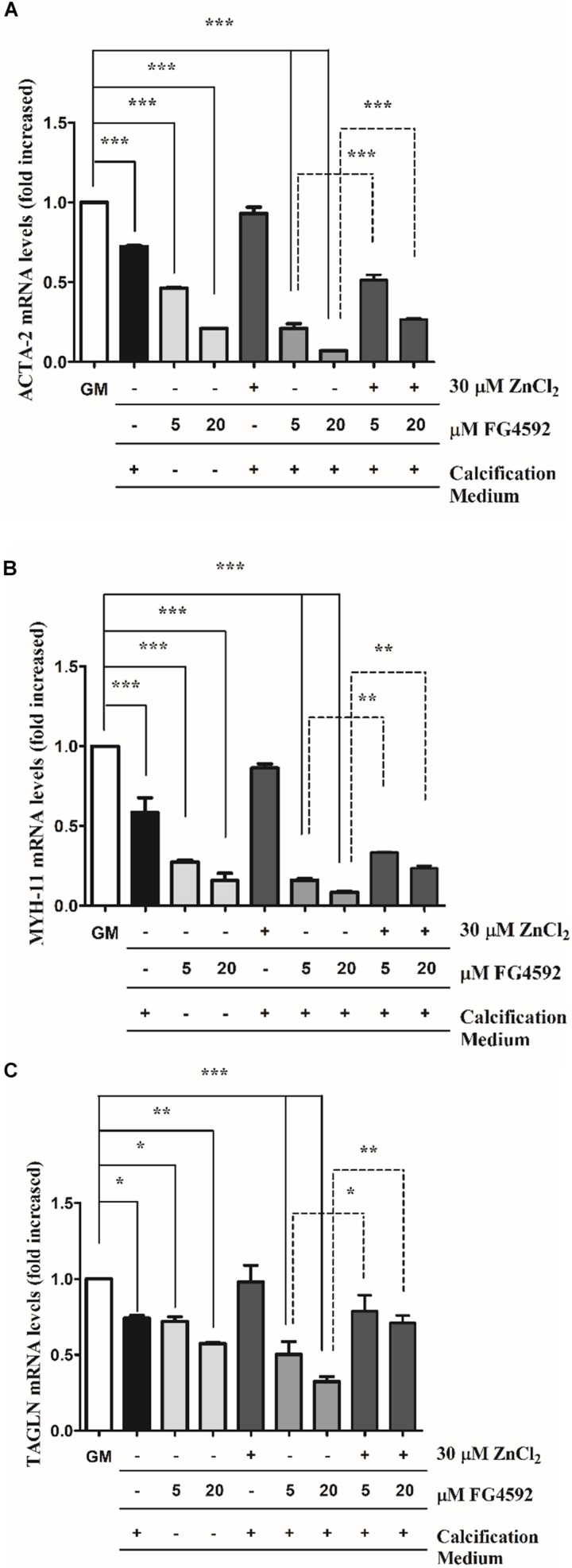
Prolyl hydroxylase inhibitor (PHI) FG4592 decreases smooth muscle marker expression that is prevented by zinc. VSMCs were cultured in growth medium in the presence or absence of PHI (5 and 20 μmol/L) or in calcification medium with or without PHI FG4592 and zinc for 10 days, and the expression of smooth muscle-specific markers were measured using qRT-PCR **(A–C)**. **(A)** Relative expression of ACTA-2 (smooth muscle alpha (α)-2 actin), **(B)** MYH11 (smooth muscle myosin heavy chain 11), and **(C)** TAGLN (smooth muscle protein 22-α) were determined by qRT-PCR and normalized to RNA45S5. Results are presented as mean ± SEM of three independent experiments. Statistical analysis was performed by one-way ANOVA test followed by Bonferroni correction. A value of *p* < 0.05 was considered significant. ^∗^*p* < 0.05, ^∗∗^*p* < 0.01, ^∗∗∗^*p* < 0.001.

Downregulation of smooth muscle-specific markers is a common characteristic of VSMCs calcification (Steitz et al., 2001), and recent evidence shows that HIF1α induces phenotypic switch in VSMCs (Liu et al., 2017). Therefore, we examined whether PHI aggravates Pi-induced loss of smooth muscle-specific markers such as smooth muscle α-actin (ACTA-2), smooth muscle myosin heavy chain 11 (MYH11), and smooth muscle protein 22α (TAGLN). VSMCs were cultured in growth medium in the presence or absence of PHI (5 and 20 μmol/L) or in calcification medium with or without PHI and zinc for 3, 6, and 10 days. We showed that Pi induced the downregulation of ACTA-2 (Figure 3A), MYH-11 (Figure 3B), and TAGLN (Figure 3C) expression that was significantly aggravated by PHI in a dose-dependent manner after 10 days. These results support the hypothesis that high Pi and PHI synergistically boost calcification of VSMCs. Interestingly, PHI also induced a dose-dependent decrease in smooth muscle markers in growth medium without high Pi (Figures 3A-C). Importantly, zinc (30 μmol/L) significantly restored smooth muscle-specific gene expression in response to Pi as well as to Pi + PHI (Figures 3A–C). In addition, time-course experiments showed that the increased loss of smooth muscle-specific markers in response to PHI can be detected as early as 3 days, which is also attenuated by zinc (Supplementary Figures 1A–F). Overall, these data suggest that PHI downregulates smooth muscle-specific markers independently of the Pi level, however, Pi and PHI act synergistically to decrease smooth muscle-specific markers, which is attenuated by zinc.

### PHI Enhances the Expression of Osteochondrogenic Markers That Is Inhibited by Zinc

**FIGURE 4:**
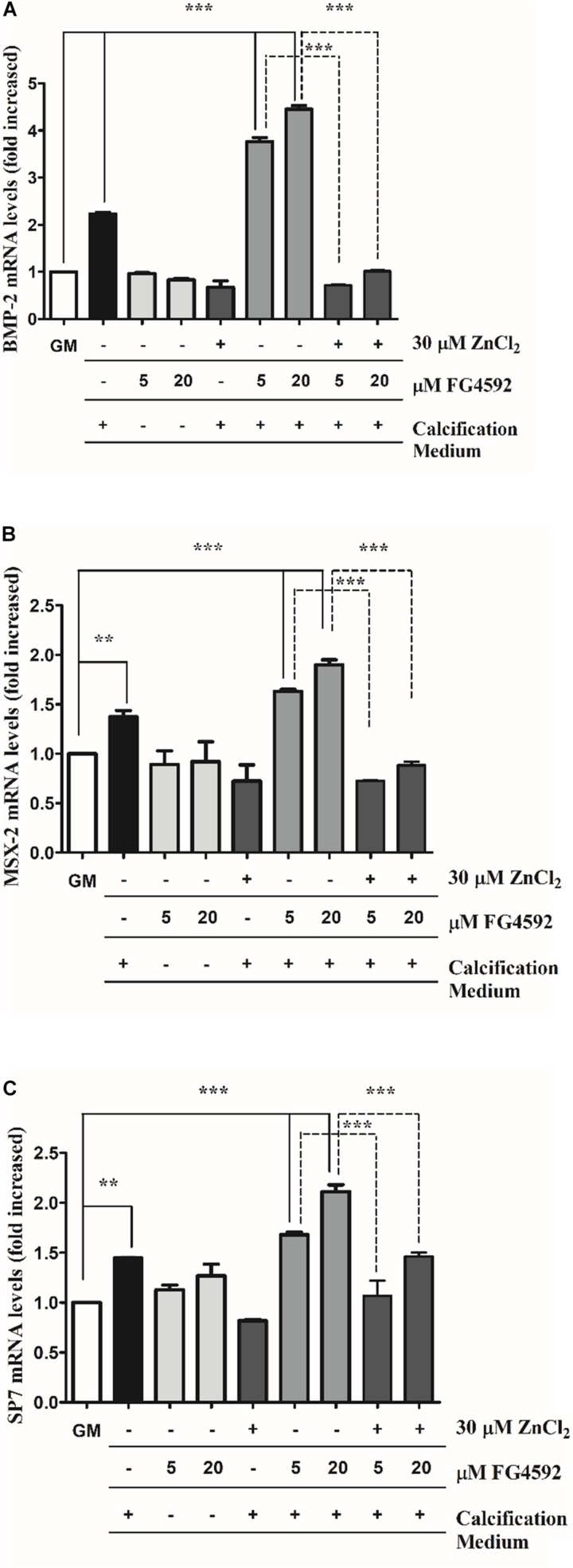
Prolyl hydroxylase inhibitor (PHI) FG4592 enhances the expression of osteochondrogenic markers that is inhibited by zinc. VSMCs were cultured in growth medium in the presence or absence of PHI FG4592 (5 and 20 μmol/L) or in calcification medium with or without PHI and zinc for 10 days, and the expression of osteochondrogenic markers was measured using qRT-PCR **(A–C)**. **(A)** Relative expression of BMP-2 (bone morphogenic protein-2), **(B)** Msx-2 (Msh Homeobox 2), and **(C)** SP7 were determined by qRT-PCR and normalized to RNA45S5. Results are presented as mean ± SEM of three independent experiments. Statistical analysis was performed by one-way ANOVA test followed by Bonferroni correction. A value of *p* < 0.05 was considered significant. ^∗^*p* < 0.05, ^∗∗^*p* < 0.01, ^∗∗∗^*p* < 0.001.

To decipher the molecular mechanism involved in PHI-enhanced calcification of VSMCs induced by Pi, and to explore zinc’s inhibitory effect on this process, a possible direct effect of PHI on osteochondrogenic gene expressions of VSMCs were investigated in time-course experiments (Figures 4A–C and Supplementary Figures 2A–F). High Pi increased BMP-2, Msx-2 as well as Sp7 expression after 10 days that was aggravated by PHI (Figures 4A–C). These effects were significantly reduced by zinc supplementation. Importantly, PHI did not influence osteochondrogenic gene expression without high Pi level even after 10 days (Figures 4A–C). We showed that neither high Pi nor PHI and high Pi + PHI induced BMP-2 and Msx-2 expression as early as 3 or 6 days (Supplementary Figures 2A–D), and only Sp7 gene expression was elevated at 6 days in response to Pi + PHI (20 μmol/L), which was ameliorated by zinc. Taken together, PHI aggravated osteochondrogenic gene expression induced by high Pi, which was ameliorated by zinc.

### PHI Influences Ser451 Phosphorylation of Runx2 and Induces Pyruvate Dehydrogenase Kinase 4 Expression

**FIGURE 5:**
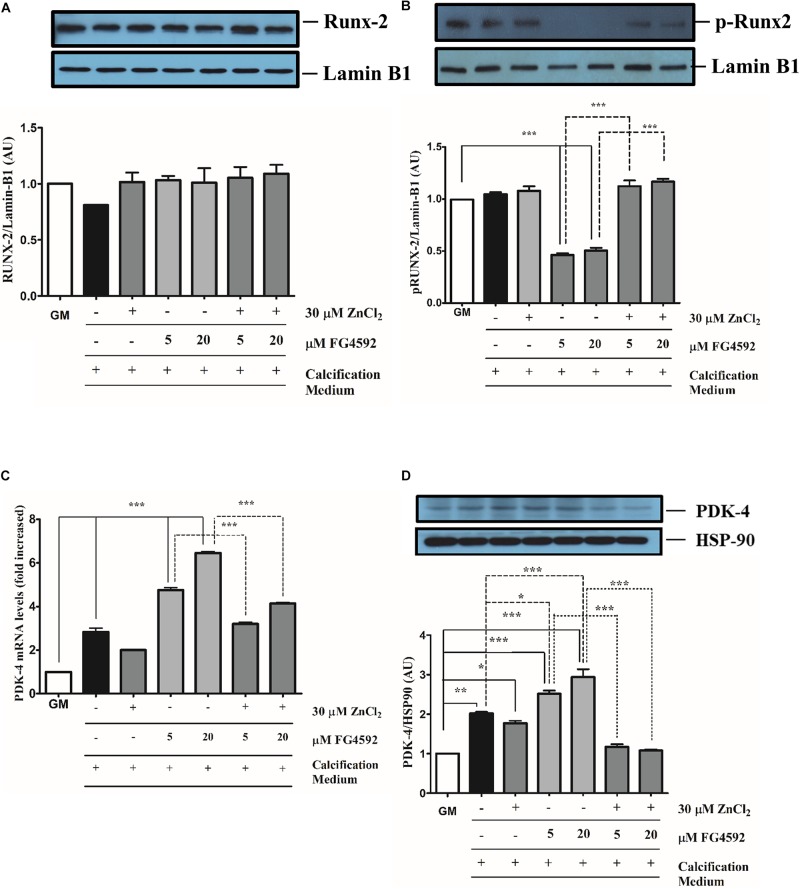
Prolyl hydroxylase inhibitor (PHI) FG4592 influences Ser451 phosphorylation of Runx2 and induces pyruvate dehydrogenase kinase 4 expression VSMC cultured in calcification medium were treated with PHI FG4592 (5 and 20 μmol/L, respectively) in the presence (30 μmol/L) or absence of zinc for 10 days **(A–D)**. **(A)** Nuclear translocation and **(B)** Ser451 phosphorylation of Runx2 were analyzed by immunoblot of nuclear extracts followed by the densitometric analyses of blots from three independent experiments. Results are presented as mean ± SEM of three independent experiments normalized to Lamin B1. Statistical analysis was performed by one-way ANOVA test followed by Bonferroni correction. A value of *p* < 0.05 was considered significant. ^∗^*p* < 0.05, ^∗∗^*p* < 0.01, ^∗∗∗^*p* < 0.001. Blots show one representative image from three independent experiments. **(C)** The expression of PDK4 was analyzed by qRT-PCR and normalized to RNA45S5. Results are presented as mean ± SEM of three independent experiments. Statistical analysis was performed by one-way ANOVA test followed by Bonferroni correction. A value of *p* < 0.05 was considered significant. ^∗^*p* < 0.05, ^∗∗^*p* < 0.01, ^∗∗∗^*p* < 0.001. **(D)** The expression of PDK4 was analyzed by immunoblot and normalized to Hsp90. Results are presented as mean ± SEM of three independent experiments normalized to Lamin B1. Statistical analysis was performed by one-way ANOVA test followed by Bonferroni correction. A value of *p* < 0.05 was considered significant. ^∗^*p* < 0.01, ^∗∗∗^*p* < 0.001, ^∗∗∗^*p* < 0.001. Blots show one representative image from three independent experiments.

Runx2-dependent transactivation of osteochondrogenic genes is greatly influenced by the phosphorylation of its serine residues (Wee et al., 2002; Ge et al., 2009; Li et al., 2017). To gain further insight into the mechanism by which zinc inhibits Pi and Pi + PHI mediated calcification of VSMC, we analyzed the nuclear translocation and phosphorylation of Runx2 Ser451 in nuclear extracts derived from cells treated with Pi and Pi + PHI in the presence or absence of zinc (Figures 5A,B and Supplementary Figures 3A,B). As a result, we showed that the nuclear translocation of Runx2 was not affected either by Pi or Pi + PHI after 10 days (Figure 5A). We next explored whether Pi or PHI + Pi influences Ser451 phosphorylation after 3, 6, and 10 days. The phosphorylation level of Runx2 Ser451 was unaltered in cells exposed to high Pi alone at all time points but was significantly reduced after 6 and 10 days when PHI was also present in the calcification medium (Figure 5B and Supplementary Figure 3B). The phosphorylation of Runx2 Ser451 was unaltered after 3 days in response to high Pi + PHI (Supplementary Figure 3A). Importantly, zinc restores the phosphorylation level of Ser451 in response to high Pi + PHI after 6 and 10 days of treatments (Figure 5B and Supplementary Figure 3B).

**FIGURE 6:**
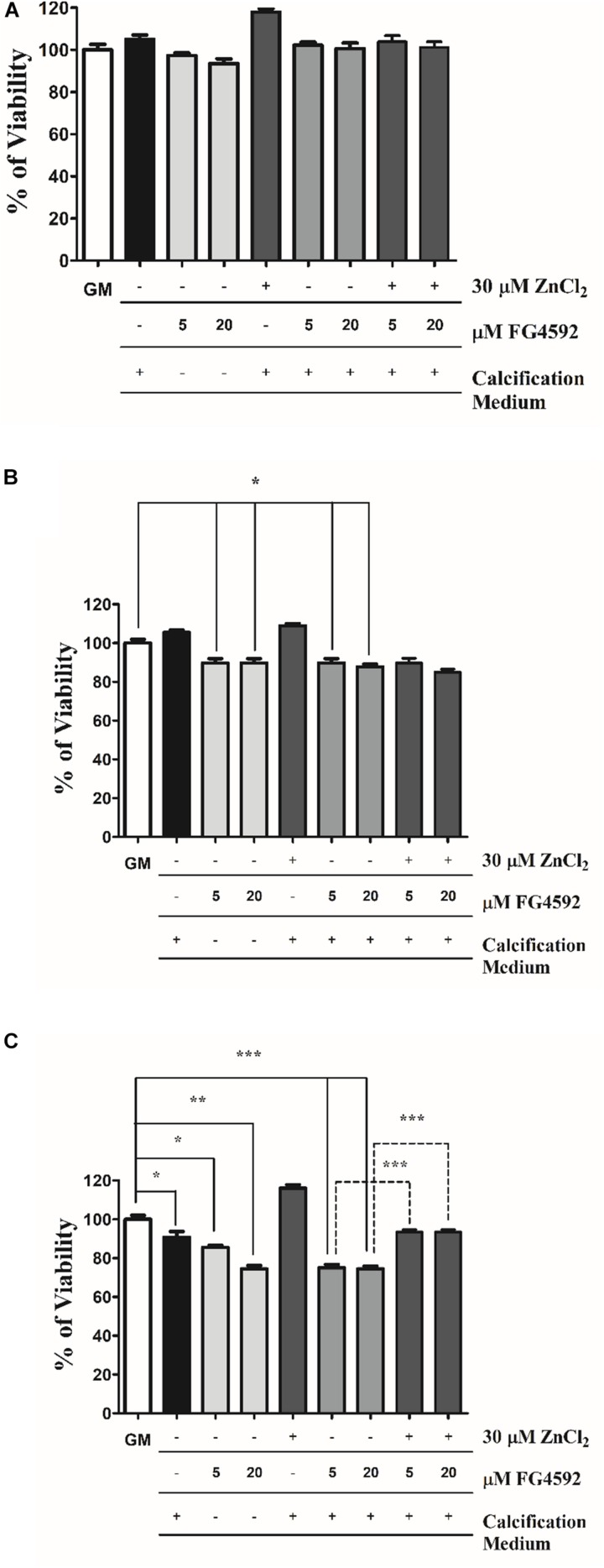
Zinc restores the viability of VSMCs in response to high Pi and PHI FG4592. VSMCs were cultured in growth medium in the presence or absence of PHI FG4592 (5 and 20 μmol/L) or in calcification medium with or without PHI FG4592 and zinc for 3, 6, and 10 days. **(A–C)**. Cell viability was determined by MTT assay after **(A)** 3, **(B)** 6, and **(C)** 10 days. Statistical analysis was performed by one-way ANOVA test followed by Bonferroni correction. A value of *p* < 0.05 was considered significant. ^∗^*p* < 0.05, ^∗∗^*p* < 0.01, ^∗∗∗^*p* < 0.001.

Pyruvate dehydrogenase kinase 4 plays an important role in vascular calcification (Lee et al., 2015; Zhu et al., 2018). We next explored whether PDK4 is involved in the enhanced calcification triggered by PHI in calcification medium (Figures 5C,D). Pi alone led an about 3-fold induction in PDK4 mRNA measured by reverse transcription qPCR after 10 days which was further increased by PHI in a dose-dependent fashion (Figure 5C). Elevated PDK4 mRNA levels were significantly attenuated when zinc (30 μmol/L) was added to the Pi medium with or without PHI (Figure 6C). Consistent with these findings, PDK4 protein levels were also increased by PHI which was also mitigated by zinc (Figure 5D). These data demonstrate that both transcriptional and post-translational effects of PHI on VSMC calcification can be counteracted by zinc.

### Zinc Restores the Viability of VSMCs in Response to High Pi and PHI

It has been reported that zinc improves cell viability during VSMCs calcification (Shin and Kwun, 2014). To explore the involvement of cell death in PHI-aggravated calcification, VSMCs were treated with PHI in growth medium alone and or in calcification medium in the presence or absence of PHI and zinc for 3, 6, and 10 days. As illustrated in Figure 6, neither high Pi nor PHI and Pi + PHI affected cell viability after 3 days (Figure 6A). However, after 6 days, PHI decreased cell viability independently of the presence of Pi that was not affected by zinc (Figure 6B). After 10 days, cell viability was decreased in response to high Pi, which was aggravated by PHI (Figure 6C). Similar to the 6-day-treatments, PHI induced cell death regardless the Pi level in the experimental medium. In contrast to the 6-day-experiments, zinc restored cell viability after 10 days (Figure 6C). Thus, zinc prevents the decrease in cell viability in response to Pi + PHI only in the late phase of the experiments.

### Inhibitory Effect of Zinc Is Independent of Zinc-Responsive Protein Metallothionein

Metallothioneins (MTs) are abundantly induced by a high amount of zinc and cadmium (Hamer, 1986) and characterized by potent antioxidant properties (Viarengo et al., 2000). Since reactive oxygen species (ROS) and oxidative stress are linked to vascular calcification (Scholze et al., 2016), next we examined whether zinc inhibits Pi provoked mineralization of VSMC in an MT-dependent fashion. VSMC were cultured in calcification medium containing 3 mmol/L Pi in the presence (30 μmol/L), or absence of zinc for 10 days and MT1/MT2 gene expressions were analyzed by qRT-PCR. Our results showed that exposure of cells to zinc (30 μmol/L), slightly above the physiological concentration, did not induce MT1a and MT2a in our experimental conditions compared to untreated cells (Data not shown).

**FIGURE 7:**
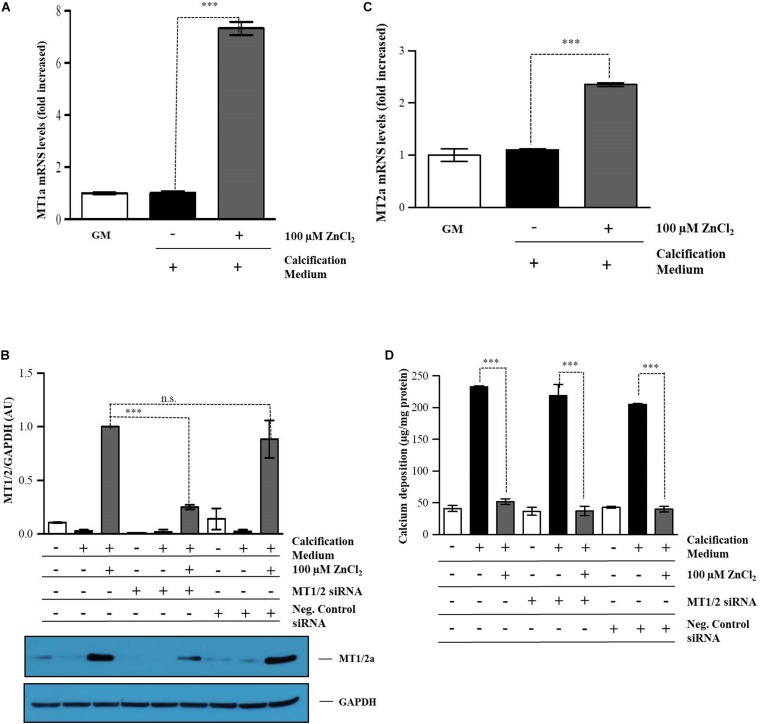
Inhibitory effect of zinc is independent of zinc-responsive protein metallothionein. VSMCs were cultured in calcification medium containing 3 mmol/L Pi in the presence (100 μmol/L), or absence of zinc for 10 days **(A,B)**. Relative expression of methallothionenin 1A **(A)** and 2A **(B)** were determined by qRT-PCR and normalized to RNA45S5. Results are presented as mean ± SEM of three independent experiments. Statistical analysis was performed by one-way ANOVA test followed by Bonferroni correction. A value of *p* < 0.05 was considered significant. ^∗^*p* < 0.05, ^∗∗^*p* < 0.01, ^∗∗∗^*p* < 0.001. VSMCs were transfected with MT1a/2A siRNA and cultured in calcification medium containing 3 mmol/L Pi in the presence (100 μmol/L), or absence of zinc for 10 days. Gene expression of MTs was analyzed by q-RT-PCR and immunoblot after 10 days. **(C,D)**. **(C)** MT1A/2A expression was analyzed by immunoblot. Gene expression of MTs was analyzed by q-RT-PCR and immunoblot after 10 days. Statistical analysis was performed by one-way ANOVA test followed by Bonferroni correction. A value of *p* < 0.05 was considered significant. ^∗^*p* < 0.05, ^∗∗^*p* < 0.01, ^∗∗∗^*p* < 0.001. **(D)** Calcium content of solubilized ECM is presented in MT1A/B knocked down cells. Data are expressed as mean ± SEM of three independent experiments. Statistical analysis was performed by one-way ANOVA test followed by Bonferroni correction. A value of *p* < 0.05 was considered significant. ^∗^*p* < 0.05, ^∗∗^*p* < 0.01, ^∗∗∗^*p* < 0.001.

Metallothioneins have been shown to be expressed in atherosclerotic plaques representing a local protective mechanism (Göbel et al., 2000; Daskalopoulou et al., 2007). This prompted us to examine whether MT expression provoked by supraphysiological zinc concentration (100 μmol/L), seven-time higher than the normal plasma level, inhibits calcification of VSMC *in vitro*. Zinc (100 μmol/L) markedly increased the expression of MT1a/2a compared to either the untreated VSMCs or cells cultured in the calcification medium (Figure 7A). To explore the role of MT1a/2a on VSMC mineralization in this calcification model, MT1a/2a expression was silenced with MT1/2 specific siRNAs. Q-RT-PCR and immunoblot analysis of VSMC demonstrated that siRNA treatment effectively decreased MT1/2 expression in cells exposed to zinc (100 μmol/L) (Figure 7B). Interestingly, ECM calcium deposition was not affected by MT1a/2a levels, since zinc inhibited Pi induced calcium deposition despite suppressed MT1a/2a expression (Figures 7C,D). This observation suggests that the inhibitory effect of zinc on Pi induced mineralization is independent MT.

### Plasma Zinc Level Is Reduced in Patients Undergoing Carotid Endarterectomy and in CKD Patients on Hemodialysis

**FIGURE 8:**
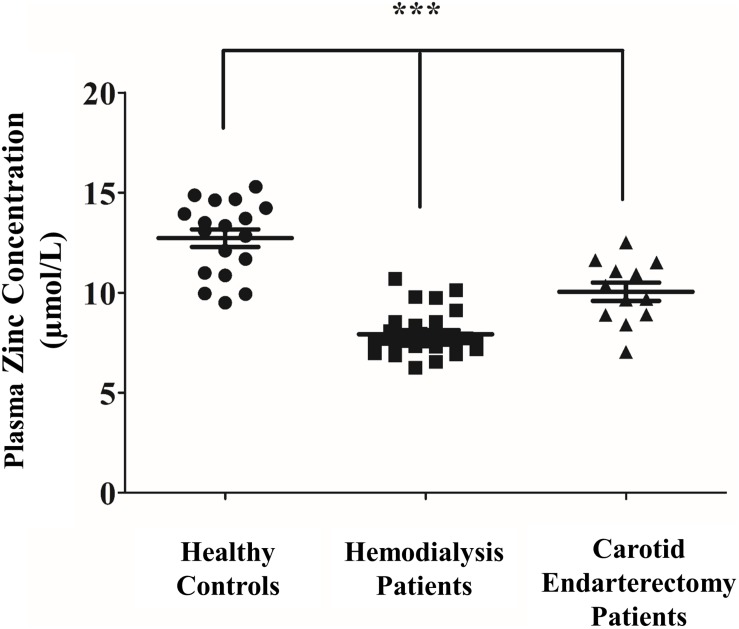
Plasma zinc level is reduced in patients undergoing carotid endarterectomy and in CKD patients on hemodialysis. Healthy volunteers without any known diseases (*n* = 18, mean age 46 years, F/M 7/11), Stage 5 CKD patients subjected to hemodialysis (*n* = 28, mean age 55 years, F/M 15/13), and patients undergoing carotid endarterectomy (*n* = 15, mean age 72 years, F/M 6/9) were recruited and plasma zinc levels were measured with inductively coupled plasma-atomic emission spectrometry (ICP-AES). Statistical analysis was performed by one-way ANOVA test followed by Bonferroni correction. A value of *p* < 0.05 was considered significant. ^∗^*p* < 0.05, ^∗∗^*p* < 0.01, ^∗∗∗^*p* < 0.001.

A recent report revealed that reduced zinc levels in CKD contribute to the severity of vascular calcification (Voelkl et al., 2018), however, the possible link between plasma zinc level and progression of vascular disease in patients with physiologic kidney function has not been established. Therefore, we asked whether low zinc plasma concentration is associated with vascular disease in patients diagnosed with significant carotid artery stenosis undergoing carotid endarterectomy. Plasma zinc levels were measured with inductively coupled plasma-atomic emission spectrometry (ICP-AES) in patients with intact kidney function undergoing carotid surgery and compared to CKD patients on hemodialysis and healthy controls (Figure 8). According to previous studies, plasma zinc levels were reduced in CKD patients compared to healthy subjects. Importantly, plasma zinc levels of patients with carotid artery stenosis were significantly reduced compared to healthy controls. These data suggest that low plasma zinc concentration is a potential risk factor for vascular diseases of different etiology.

## Discussion

The results of this study demonstrate that HIF PHI aggravates high Pi-induced osteochondrogenic transformation and mineralization of VSMCs, which is inhibited by zinc supplementation. Our data highlight several key findings: (1) PHI FG4592 induces the loss of SMC-specific markers but does not trigger osteochondrogenic marker expression alone at physiologic Pi level; (2) PHI FG4592 aggravates high Pi-induced phenotypic switch of VSMCs by further repressing VSMCs marker genes and upregulating osteochondrogenic genes; (3) PHI FG4592 exacerbates VSMCs mineralization; (4) PHI FG4592 influences Ser451 phosphorylation of Runx2 and increases PDK4 expression in response to high Pi; (5) zinc inhibits osteochondrogenic reprogramming and mineralization of VSMCs in response to PHI FG4592 and high Pi; and (6) zinc plasma levels are significantly lower in CKD and in patients underwent carotid endarterectomy compared to healthy controls.

A novel therapeutic approach to correct CKD-associated anemia is the induction of EPO synthesis by stabilizing hypoxia-inducible factors (HIFs) with prolyl hydroxylase inhibitors (PHIs). PHIs are all promising candidates with great therapeutic potential for improving anemia in CKD (Carney, 2016; Gupta and Wish, 2017). However, recent reports have shown that HIFs accelerate vascular calcification (Mokas et al., 2016; Zhu et al., 2018), one of the major risk factors for morbidity and mortality in CKD (Sigrist et al., 2007; Collins et al., 2015; Schlieper et al., 2016) due to hyperphosphatemia (Hruska et al., 2008). Therefore, we investigated whether HIF PHI FG4592 triggers the osteochondrogenic differentiation of VSMCs in response to high Pi.

Growing evidence suggests that hypoxia and HIFs play a vital role in vascular calcification, and targeting HIF blocks mineralization in hypoxia (Mokas et al., 2016; Balogh et al., 2019). Our results presented here support the link between HIFs and vascular calcification. We demonstrate that the pharmacologic stabilization of HIFs by PHI aggravates high Pi-induced mineralization of VSMCs, which indicates that PHIs, due to their HIF-stabilizing effect, might accelerate vascular calcification in CKD.

Another link between HIFs and vascular calcification has recently been suggested by Zhu et al. (2018). This study demonstrates that the stabilization and nuclear translocation of HIF accelerate VSMC calcification *via* PDK4 in VSMCs, while inhibition of PDK4 attenuates mineralization. Others have shown that PDK4 expression is increased in calcifying VSMCs as well as in the vessel walls of patients suffering from vascular calcification (Lee et al., 2015). Our data demonstrate that the stabilization of HIFs by PHI also induces PDK4 expression, which is likely to be involved in the accelerated calcification of VSMCs in response to high Pi and PHI FG4592.

Osteochondrogenic reprogramming of VSMCs is a typical phenomenon during mineralization. Elevated extracellular Pi induces phenotypic switch of VSMCs into osteoblast-like cells enhancing vascular calcification by reducing the expression of VSMC markers (smooth muscle 22α, SM22α; smooth muscle alpha-actin, SM α-actin) and up-regulating osteoblast-specific genes (Steitz et al., 2001). In line with these observations, we confirmed that high extracellular Pi induces osteochondrogenic genes and down-regulates smooth muscle markers. A recent report has shown that HIF-1α triggers phenotypic switch in VSMCs by down-regulating smooth muscle cell-specific genes (Liu et al., 2017). Similar to this report, here we show that by stabilizing HIF, PHI FG4592 down-regulates smooth muscle-specific gene expression. Importantly, this effect was also observed at the physiological Pi level. This is in agreement with the finding of Nishide et al. (2019) who have shown that PHI FG4592 can alter the phenotype of tumor-infiltrating macrophages. Our results also demonstrate that high Pi and PHI FG4592 synergistically lower SMC-specific gene levels in VSMCs suggesting that CKD patients might be at greater risk to develop vascular calcification in response to PHI FG4592 therapy due to their elevated Pi levels.

Osteochondrogenic transformation of VSMCs also involves proteins regulating osteoblast development in bones play a pivotal role in the osteoblastic transformation and calcification of VSMCs. BMP-2 is commonly expressed in calcified human atherosclerotic plaques (Boström et al., 1993) and dose-dependently triggers phosphate-induced calcification in human VSMC (Li et al., 2008). It has been reported that hypoxia induces BMP-2 expression in osteoblasts (Tseng et al., 2010) and microvascular endothelial cells (Bouletreau et al., 2002). Our data presented here demonstrate that PHI FG4592 exacerbates Pi-induced expression of osteochondrogenic genes such as BMP-2, Msx-2, and Sp7. Our findings are in good agreement with other studies demonstrating that hypoxia increases osteogenic gene expression in mesenchymal stem cells (Ding et al., 2014) and periosteal cells (Ichijima et al., 2012) which suggest this mode of action by which PHI FG4592 enhances Pi-induced calcification of VSMCs. Interestingly, PHI FG4592 at physiologic Pi level does not induce osteochondrogenic gene expression suggesting that PHIs do not accelerate vascular calcification at the normal Pi level.

Runx2-dependent activation of osteochondrogenic gene expression is affected by the post-transcriptional modifications of Runx2 (Wee et al., 2002; Ge et al., 2009; Li et al., 2017). Phosphorylation of different serine (S) residues greatly affects its transactivation properties and ubiquitin-dependent degradation. S451 phosphorylation could act to downregulate Runx2-dependent transactivation (Wee et al., 2002). Here we show that PHI FG4592 decreases the phosphorylation of S451 in VSMCs in response Pi. However, phosphorylation of S451 is not influenced by the calcification medium alone. This suggests that the decreased phosphorylation level of Runx2 S451 might be involved in the more rapid and extensive calcification as well as the robust induction in osteochondrogenic genes provoked by PHI FG4592 compared to high Pi alone. Importantly, zinc restores the phosphorylation level of Runx2 Ser451 that might be a possible mechanistic mode of action by which zinc inhibits osteochondrogenic gene activation by Runx2.

Recent reports have shown that zinc attenuates vascular calcification (Shin and Kwun, 2014; Voelkl et al., 2018) by restoring VSMC viability (Shin and Kwun, 2014). Our results support the protective effect of zinc in vascular calcification by enhancing VSMC viability in response to PHI and high Pi.

Evidence suggests that ROS and oxidative stress are involved in the pathogenesis of vascular calcification (Byon et al., 2008; Agharazii et al., 2015) that is effectively ameliorated by zinc supplementation *in vitro* (Shin and Kwun, 2013, 2014, 2016; Voelkl et al., 2018). One tenable hypothesis is that zinc may induce ROS scavenger proteins which are able to reduce calcification by scavenging ROS. Metallothioneins (MTs) are abundantly induced by high levels of zinc and cadmium (Hamer, 1986) and characterized by potent antioxidant properties (Viarengo et al., 2000). In addition, VSMCs in atherosclerotic plaques express MTs which act as scavengers for ROS (Göbel et al., 2000), but this has not been experimentally tested so far. Here we show that MTs are not involved in the protective effect of zinc against vascular calcification, and other mechanisms than MTs are responsible for the protective effect of zinc supplementation.

This link between zinc and vascular diseases is also suggested by Voelkl et al. who has shown that low zinc level might be associated with increased calcification in CKD (Voelkl et al., 2018). In accordance with this study, we detect low plasma zinc levels in dialysis dependent-CKD patients compared to healthy controls. Similar to the observations in CKD, patients who underwent carotid endarterectomy present also reduced zinc plasma levels compared to healthy controls. Our data suggest that low plasma zinc level is associated with vascular calcification not only in CKD but also in patients diagnosed with significant carotid artery stenosis. This phenomenon underlines the importance of monitoring and maintaining physiological plasma zinc levels in patients with increased risk for vascular calcification (Chen and Moe, 2012; Chen et al., 2017).

**FIGURE 9:**
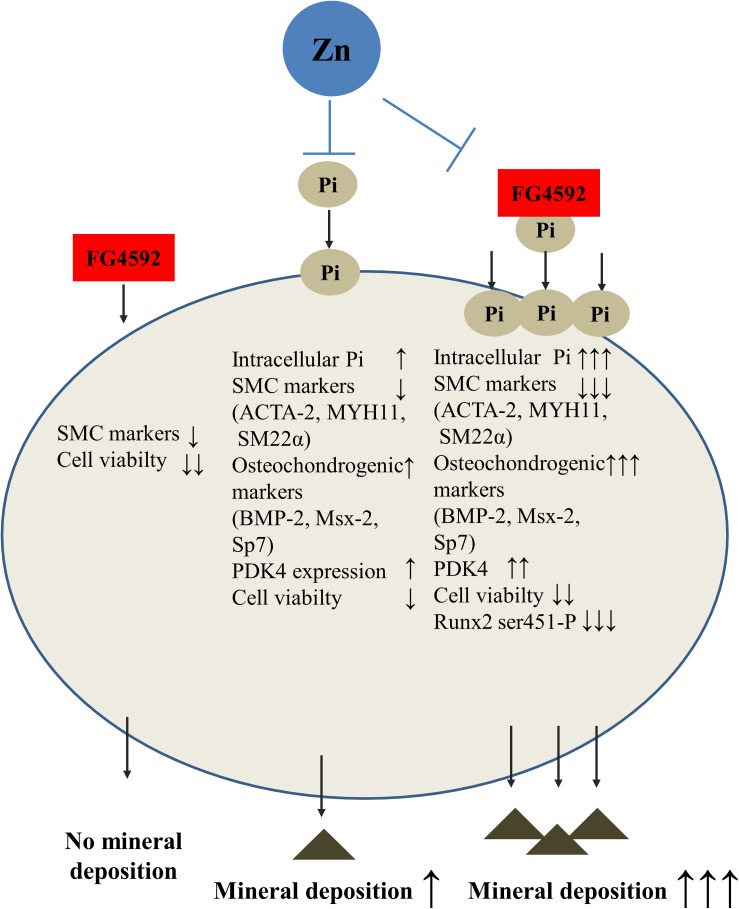
Zinc protects against phosphate- and HIF-prolyl hydroxylase inhibitor-induced vascular smooth muscle cell calcification. PHI FG4592 aggravates vascular calcification by increasing phosphate (Pi) uptake and calcium deposition by VSMCs, lowering smooth muscle-specific gene expression (ACTA-2 [smooth muscle alpha (α)-2 actin], MYH11 [smooth muscle myosin heavy chain 11], and TAGLN ([smooth muscle protein 22-α], increasing osteochondrogenic gene expression (BMP-2 [bone morphogenic protein-2], Msx-2 [Msh Homeobox 2], and SP7), inducing pyruvate dehydrogenase kinase 4 (PDK4), decreasing the phosphorylation level of Runx2 Ser451, and lowering cell viability. Importantly, zinc inhibits PHI FG4592-aggravated calcification by high Pi *via* maintaining VSMC phenotype, decreasing Pi uptake, lowering osteochondrogenic gene expression and the level of PDK4 as well as preserving Runx2 Ser451 phosphorylation and cell viability.

Here we demonstrate that PHI FG4592 is a potent inducer of osteogenic differentiation of VSMCs in elevated Pi environments. Zinc has the capability to prevent PHI FG4592 induced mineralization of VSMCs and osteogenic gene induction in high Pi media. The beneficial role of zinc in Pi-mediated calcification has been suggested by other studies as well (Shin and Kwun, 2016; Voelkl et al., 2018). Our results described here showed that PHI FG4592, a promising candidate for correcting anemia in CKD, markedly exacerbates Pi-induced calcification of VSMCs. PHI FG4592 promotes vascular calcification by increasing Pi uptake and calcium deposition by VSMCs, triggering their phenotypic reprogramming, inducing PDK4, and modulating the phosphorylation level of Runx2. Importantly, zinc attenuates PHI FG4592-promoted calcification by high Pi (Figure 9). These observations suggest that (1) extended follow-up period should be implicated in clinical trials with CKD patients using PHIs; (2) the pro-calcification effect of other PHIs should also be tested; and (3) zinc plasma levels should be closely monitored parallel with EPO and hemoglobin levels in patients during the administration FG4592 or other PHIs, and zinc supplementation might be considered. In addition, our data suggest that low plasma zinc level might be an independent risk factor for developing vascular calcification independently from renal disease. This necessitates the screening of plasma zinc levels in susceptible patients to reduce the risk for vascular calcification.

## Data Availability Statement

The datasets generated for this study are available on request to the corresponding author.

## Ethics Statement

The studies involving human participants were reviewed and approved by the Regional and Institutional Ethics Committee of the University of Debrecen, Medical and Health Science Center. The patients/participants provided their written informed consent to participate in this study.

## Author Contributions

AN, DP, EZ, and MN designed and performed the experiments and analyzed the data. JP performed the ICP-AES analysis. TG wrote and edited the manuscript. AZ, AA, GB, and JB revised and edited the manuscript. GB and JB designed the study.

## Conflict of Interest

The authors declare that the research was conducted in the absence of any commercial or financial relationships that could be construed as a potential conflict of interest.
