# Supplementary material for: Zinc Inhibits HIF-Prolyl Hydroxylase Inhibitor-Aggravated VSMC Calcification Induced by High Phosphate
Source: Front Physiol. 2020 Jan 15;10:1584. doi: 10.3389/fphys.2019.01584 (PMC6974455; doi:10.3389/fphys.2019.01584)
Supplement: Supplementary file 1 [file Data_Sheet_1.docx]

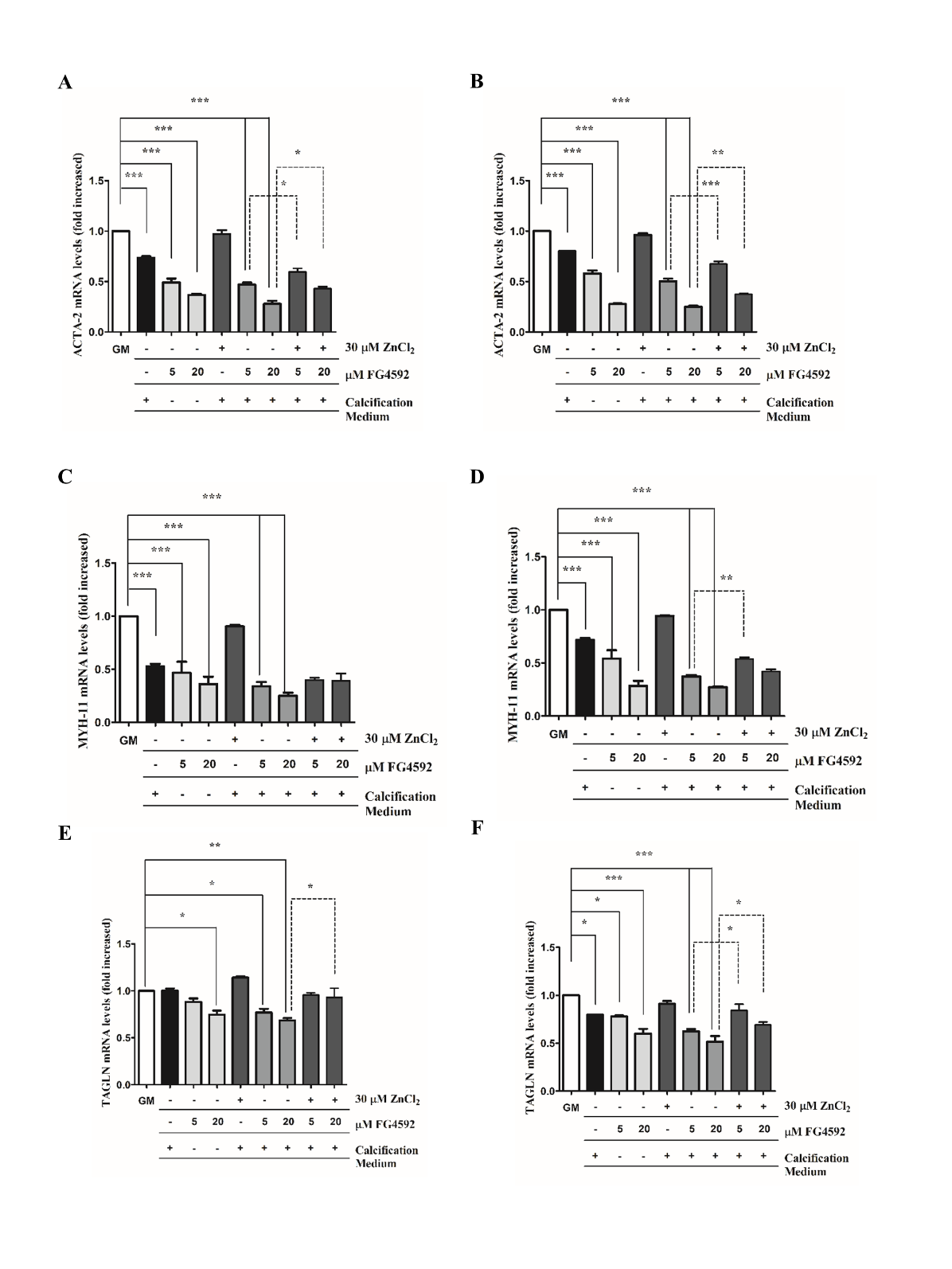


**Supplementary figure 1. Prolyl-hydroxylase inhibitor (PHI) FG4592 decreases smooth muscle marker expression.** VSMCs were cultured in growth medium in the presence or absence of PHI FG4592 (5 and 20 µmol/L) or in calcification medium with or without PHI FG4592 and zinc for 3 and 6 days, and the expression of smooth muscle-specific markers was measured using qRT-PCR (A-F). Relative expression of ACTA-2 (smooth muscle alpha (α)-2 actin) after 3 days (A) and 6 days (B), and MYH11 (smooth muscle myosin heavy chain 11) after 3 days (C), and 6 days (D), as well as TAGLN (smooth muscle protein 22-α) after 3 (E) and 6 (F) days were determined by qRT-PCR and normalized to RNA45S5. Results are presented as mean ± SEM of three independent experiments. Statistical analysis was performed by one-way ANOVA test followed by Bonferroni correction. A value of p < 0.05 was considered significant. *p<0.05, **p<0.01, ***p<0.001


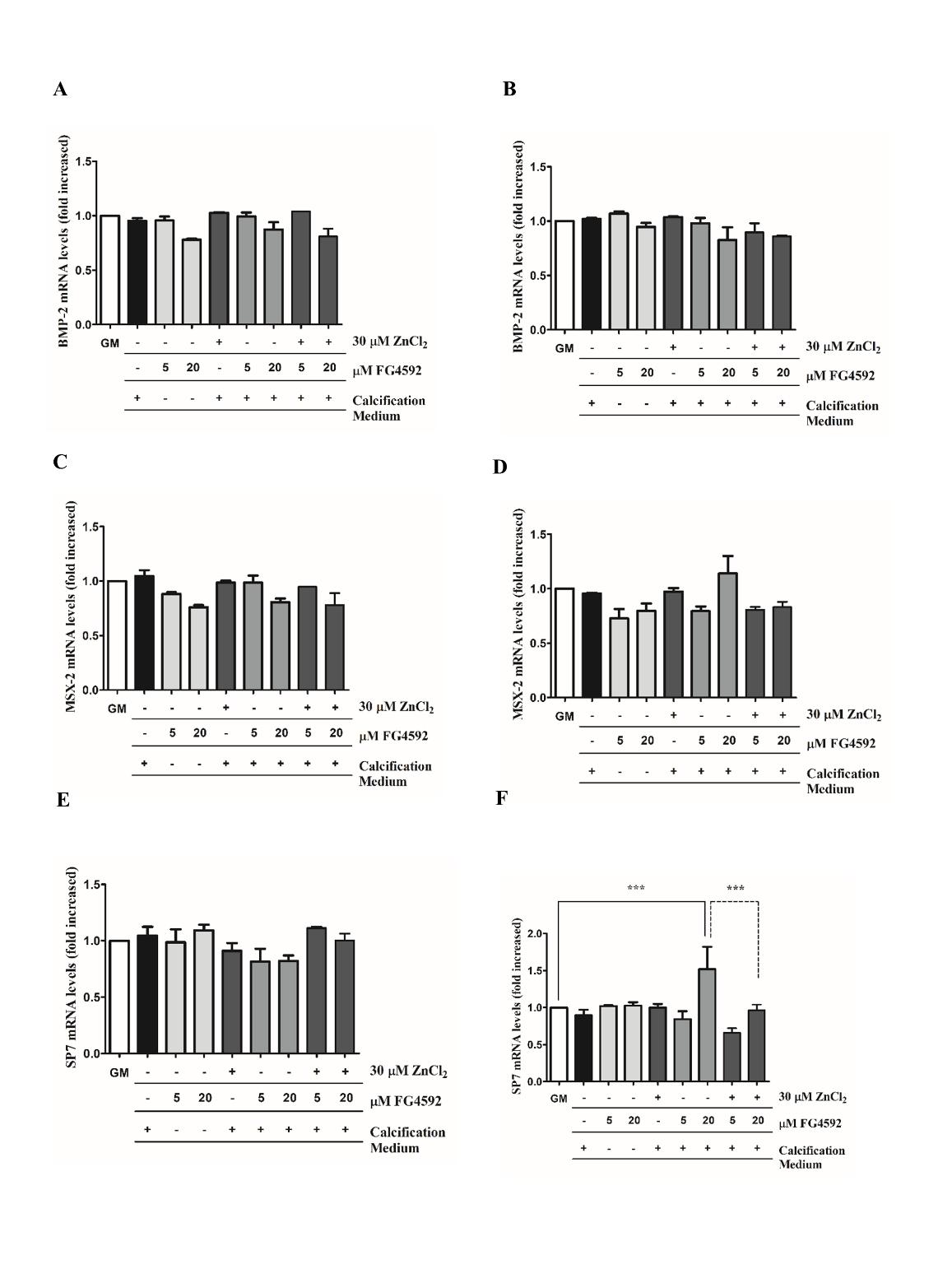


**Supplementary figure 2. Prolyl-hydroxylase inhibitor (PHI) FG4592 increases the expression of osteochondrogenic marker expression.** VSMCs were cultured in growth medium in the presence or absence of PHI FG4592 (5 and 20 µmol/L) or in calcification medium with or without PHI FG4592 and zinc for 3 and 6 days, and the expression of osteochondrogenic marker expression was measured using qRT-PCR **(A-F)**. Relative expression of BMP-2 (bone morphogenic protein-2) after 3 days **(A)** and 6 days **(B)**, and Msx-2 (Msh Homeobox 2) after 3 days **(C)**, and 6 days **(D)**, as well as SP7 after 3 **(E)** and 6 **(F)** days were determined by qRT-PCR and normalized to RNA45S5. Results are presented as mean ± SEM of three independent experiments. Statistical analysis was performed by one-way ANOVA test followed by Bonferroni correction. A value of p < 0.05 was considered significant. *p<0.05, **p<0.01, ***p<0.001


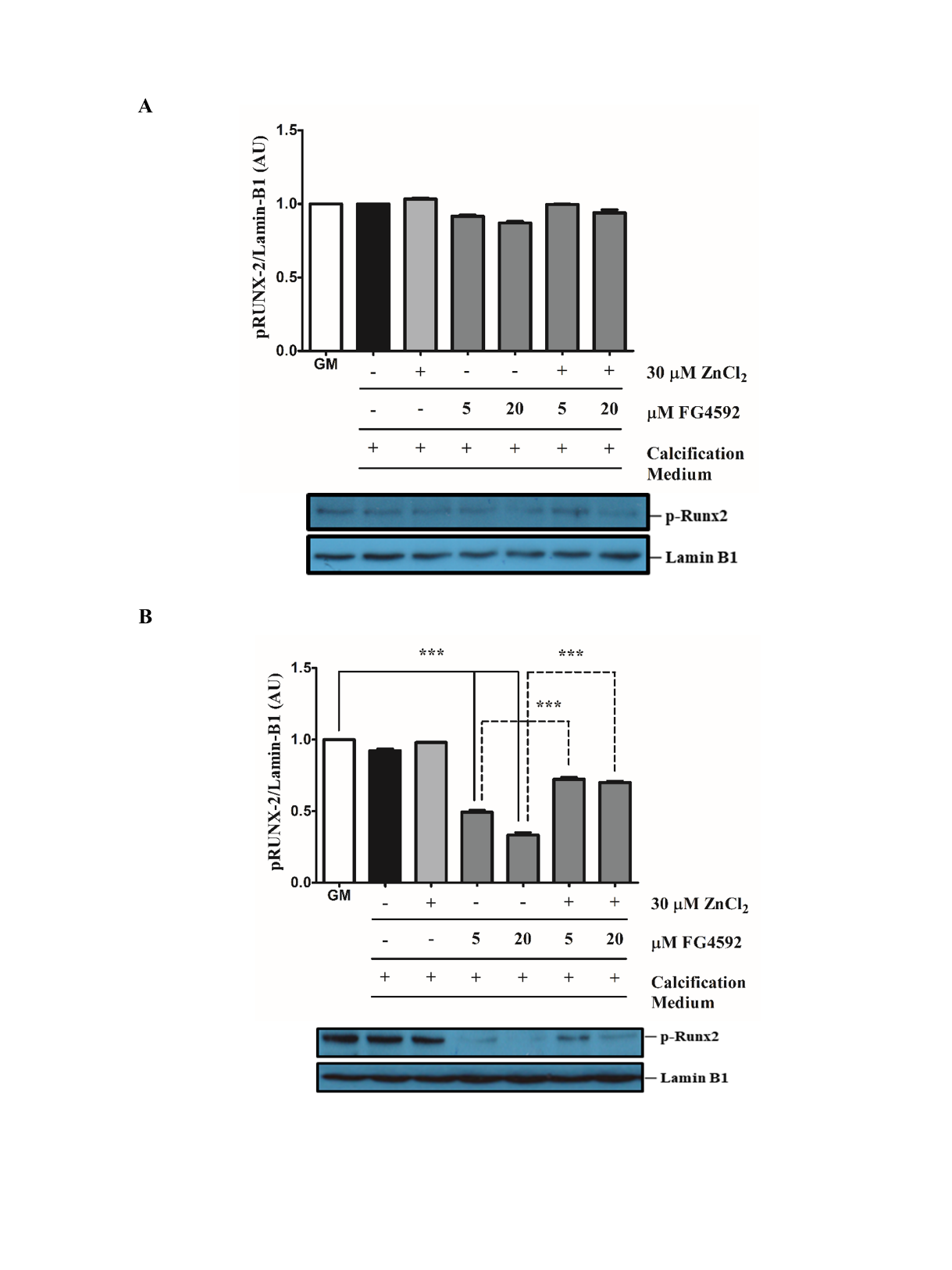


**Supplementary figure 3. Prolyl-hydroxylase inhibitor (PHI) influences the Ser451 phosphorylation of Runx2.** VSMC cultured in calcification medium were treated with PHI (5 and 20 μmol/L, respectively) in the presence (30 μmol/L) or absence of zinc for 3 or 6 days **(A-B)**. **(A)** Ser451 phosphorylation of Runx2 was analyzed by immunoblot after 3 **(A)** or 6 **(B)** days from nuclear extracts followed by the densitometric analyses of blots from three independent experiments. Results are presented as mean ± SEM of three independent experiments normalized to Lamin B1. Statistical analysis was performed by one-way ANOVA test followed by Bonferroni correction. A value of p < 0.05 was considered significant. *p<0.05, **p<0.01, ***p<0.001.
